# Change in CAIDE Dementia Risk Score and Neuroimaging Biomarkers During a 2-Year Multidomain Lifestyle Randomized Controlled Trial: Results of a Post-Hoc Subgroup Analysis

**DOI:** 10.1093/gerona/glab130

**Published:** 2021-05-10

**Authors:** Ruth Stephen, Tiia Ngandu, Yawu Liu, Markku Peltonen, Riitta Antikainen, Nina Kemppainen, Tiina Laatikainen, Jyrki Lötjönen, Juha Rinne, Timo Strandberg, Jaakko Tuomilehto, Ritva Vanninen, Hilkka Soininen, Miia Kivipelto, Alina Solomon, FINGER Study Group

**Affiliations:** 1 Institute of Clinical Medicine/Neurology, University of Eastern Finland, Kuopio, Finland; 2 Public Health Promotion Unit, Finnish Institute for Health and Welfare, Helsinki, Finland; 3 Division of Clinical Geriatrics, Center for Alzheimer Research, NVS, Karolinska Institutet, Stockholm, Sweden; 4 Department of Clinical Radiology, Kuopio University Hospital, Finland; 5 Center for Life Course Health Research/Geriatrics, University of Oulu, Finland; 6 Medical Research Center Oulu, Oulu University Hospital and Oulu City Hospital, Finland; 7 Division of Clinical Neurosciences, Turku University Hospital, Finland; 8 Turku PET Centre, University of Turku, Finland; 9 Institute of Public Health and Clinical Nutrition, University of Eastern Finland, Kuopio, Finland; 10 Joint Municipal Authority for North Karelia Social and Health Services, Joensuu, Finland; 11 Department of Public Health Solutions, National Institute for Health and Welfare Helsinki, Finland; 12 Combinostics, Tampere, Finland; 13 University of Helsinki, Clinicum, and Helsinki University Hospital, Finland; 14 Department of Public Health, University of Helsinki, Finland; 15 South Ostrobothnia Central Hospital, Seinäjoki, Finland; 16 Department of Neurosciences and Preventive Medicine, Danube-University Krems, Austria; 17 Diabetes Research Group, King Abdulaziz University, Jeddah, Saudi Arabia; 18 Neurocenter, Neurology, Kuopio University Hospital, Finland; 19 Ageing Epidemiology (AGE) Research Unit, School of Public Health, Imperial College London, UK

**Keywords:** Dementia, Hippocampus, Prevention, Risk reduction

## Abstract

The CAIDE (Cardiovascular Risk Factors, Aging and Dementia) Risk Score is a validated tool estimating dementia risk. It was previously associated with imaging biomarkers. However, associations between dementia risk scores (including CAIDE) and dementia-related biomarkers have not been studied in the context of an intervention. This study investigated associations between change in CAIDE score and change in neuroimaging biomarkers (brain magnetic resonance imaging [MRI] and Pittsburgh Compound B-positron emission tomography [PiB-PET] measures) during the 2-year Finnish Geriatric Intervention Study to Prevent Cognitive Impairment and Disability (FINGER) (post-hoc analyses). FINGER targeted at-risk older adults, aged 60–77 years, from the general population. Participants were randomized to either multidomain intervention (diet, exercise, cognitive training, and vascular risk management) or control group (general health advice). Neuroimaging (MRI and PiB-PET) data from baseline and 2-year visits were used. A toal of 112 participants had repeated brain MRI measures (hippocampal, total gray matter, and white matter lesion volumes, and Alzheimer’s disease signature cortical thickness). Repeated PiB-PET scans were available for 39 participants. Reduction in CAIDE score (indicating lower dementia risk) during the intervention was associated with less decline in hippocampus volume in the intervention group, but not the control group (Randomization group × CAIDE change interaction β coefficient = −0.40, *p* = .02). Associations for other neuroimaging measures were not significant. The intervention may have benefits on hippocampal volume in individuals who succeed in improving their overall risk level as indicated by a reduction in CAIDE score. This exploratory finding requires further testing and validation in larger studies.

Recent advances in the field of dementia prevention have highlighted the importance of modifiable risk factors (1). This provides an opportunity to intervene early, especially in individuals with higher risk of dementia. Given the multifactorial nature of dementia, no single risk factor may be sufficient for identifying people who are most likely to develop dementia. Dementia risk estimation through the use of multifactorial risk scores is a useful approach to identify individuals who may benefit most from risk reduction strategies (2). Multifactorial risk scores can be based only on nonmodifiable factors (eg, genetic risk scores) or include modifiable factors as well (3). In addition to estimating risk, the latter may also estimate the prevention potential, that is, a “room for improvement” or potential to modify the overall risk over time with dementia preventive strategies. Although risk and prevention potential are 2 sides of the same coin, most studies have so far focused on risk prediction, with far less emphasis on prevention potential.

The Cardiovascular Risk Factors, Aging and Dementia (CAIDE) Risk Score was the first midlife prediction tool combining nonmodifiable and modifiable factors. It consists of age, education, blood pressure, cholesterol, body mass index (BMI), and physical activity, and based on the midlife risk profile, it provides a 20-year dementia risk estimate (4). From a risk prediction perspective, the CAIDE score has been tested in general (5–7) and memory clinic (8) populations, and has been associated with dementia (4,5), cognitive impairment (9,10), neuroimaging measures of gray matter (GM) atrophy and white matter lesion (WML) (6,11,12), and vascular brain pathology at autopsy (13). From a prevention potential perspective, the CAIDE score seemed to work well as a potential surrogate outcome in multidomain lifestyle trials when assessing intervention effects on change in overall dementia risk (14). However, no studies have yet investigated longitudinal associations of change in CAIDE score with changes in dementia-related biomarkers in the context of prevention trials.

The Finnish Geriatric Intervention Study to Prevent Cognitive Impairment And Disability (FINGER) is the first large, longer-term randomized controlled trial to show significant benefits on cognition for a 2-year multidomain lifestyle intervention in older individuals at risk of dementia (15). The FINGER intervention also significantly reduced the estimated risk of dementia measured by the change in CAIDE score (16). The aim of the present study was to investigate associations between the change in CAIDE score and changes in brain volumes, cortical thickness, and WML volume on magnetic resonance imaging (MRI), and brain amyloid load on Pittsburgh Compound B (PiB)-positron emission tomography (PET) scans during the 2-year FINGER trial (post-hoc analyses).

## Method

### Study Design

The 2-year multidomain randomized controlled trial (FINGER) was conducted in 6 sites in Finland, enrolling an at-risk segment of the general population. The protocol (17) and primary findings (15) of the FINGER trial have been previously published. The FINGER trial (ClinicalTrials.gov identifier NCT01041989) was approved by the Coordinating Ethics Committee of the Hospital District of Helsinki and Uusimaa. All participants gave written informed consent at the screening and baseline visits and the participants in the neuroimaging subsamples gave separate consent for MRI and PiB-PET scans.

### Participants

**Figure 1.**
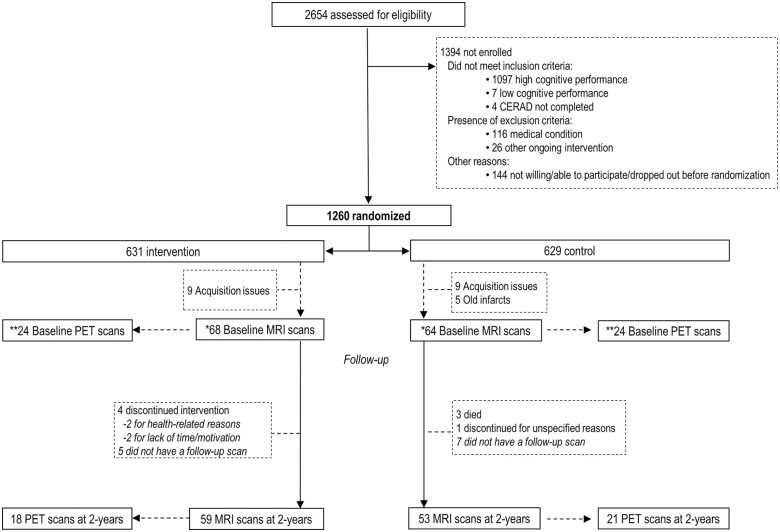
CONSORT diagram neuroimaging substudy in the FINGER trial. *Exploratory MRI outcome in a subsample at 4 trial sites (individuals [*n* = 155] most recently recruited at the time when MRI resources became available at a specific site, and with no contraindications). **Exploratory PET outcome in a subsample at 1 trial site (individuals [*n* = 48] with no contraindications). CERAD = Consortium to Establish a Registry for Alzheimer’s Disease; FINGER = Finnish Geriatric Intervention Study to Prevent Cognitive Impairment and Disability; MRI = magnetic resonance imaging; PET = positron emission tomography.

This exploratory substudy included 112 of the 1 260 FINGER participants with brain MRI scans available at both baseline and the 2-year visit, and 39 participants who had both baseline and 2-year PiB-PET scans. The FINGER neuroimaging substudy was exploratory and conducted at 4 out of 6 trial sites. Study design and protocol including CONSORT flowchart were previously described in detail (12) (also in Figure 1). Participants were the most recently recruited individuals at the time when neuroimaging resources became available at each site, and with no contraindications for MRI/PET.

FINGER comprised 1 260 individuals recruited between September 7, 2009, and November 24, 2011 from previous population-based observational cohort studies (15,17). For inclusion, the participants had to be 60–77 years old and at increased risk of dementia, that is, ≥6 points on the CAIDE score (4), and with performance on the Consortium to Establish a Registry for Alzheimer’s Disease neuropsychological battery indicating cognitive performance at the mean level or slightly lower than expected for age according to the Finnish population norms (17). Individuals having substantial cognitive impairment, dementia, conditions affecting safe participation/cooperation, or those concurrently participating in another trial were excluded.

### Randomization and Masking

Study participants were randomly assigned either to intensive multidomain intervention group, or to regular health advice (ie, control) group. The allocations were computer-generated in blocks of 4 (2 individuals randomly allocated to each group) at each site. Group allocation was not actively disclosed to participants. Outcome assessors were blinded to group allocation, and they were not involved in intervention-related activities.

### Procedures

Participants in the intervention group received 4 domains of intervention (17). The *nutrition component*, based on the Finnish Nutrition Recommendations (18), included individual and group sessions supervised by study nutritionists. The *exercise component* followed international guidelines and included gym sessions and aerobic exercise led by study physiotherapists (17). *Cognitive training* was guided by psychologists and included group sessions and computer-based individual training (web-based in-house developed program including tasks adapted from previous protocols) (19). *Management of metabolic and vascular risk factors* was conducted following national evidence-based guidelines (17). The control group received regular health advice following established guidelines (17).

### Calculation of CAIDE Dementia Risk Score

The CAIDE score (4) was calculated using data on age, sex, self-reported years of formal education, systolic blood pressure, BMI, total cholesterol, and physical activity at the baseline and 2-year visits. Table 1 describes how each risk factor in the CAIDE score was assessed, and the predefined number of points assigned to each risk factor category. CAIDE score for each FINGER participant was calculated by summing the number of points for the appropriate category of each of the risk factors.

**Table 1.** Assessment of the CAIDE Dementia Risk Score in the FINGER Trial

CAIDE Factors	Points	Measurements
Age		
<47 y	0	Population register
47–53 y	3	
>53 y	4	
Sex		
Women	0	Population register
Men	1	
Education		
≥10 y	0	Self-reported
7–9 y	2	
0–6 y	3	
Systolic blood pressure		
≤140 mm Hg	0	Trained study nurses measured blood pressure with a validated automatic device (Microlife WatchBP Office) with the participant in a sitting position, using the right arm, after 10 min of rest. The mean value of 2 measurements was used.
>140 mm Hg	2	
BMI		
≤30 kg/m^2^	0	Trained study nurses measured height (without shoes) to the nearest 0.1 cm, and weight (in light clothing). BMI was calculated by dividing the weight in kilograms by the squared height in meters.
>30 kg/m^2^	2	
Serum total cholesterol		
≤6.5 mmol/L	0	Fasting venous blood samples were taken, and total serum cholesterol was determined enzymatically using commercial reagents from Abbott Laboratories on a clinical chemistry analyzer, Architect c8000 (Abbott Laboratories, Abbott Park, IL).
>6.5 mmol/L	2	
Physical activity		
Active	0	Self-reported leisure-time physical activity was assessed with the question “How often do you participate in leisure-time physical activity that lasts at least 20–30 minutes and causes breathlessness and sweating?”. Response options were as follows: 1 = 5 times a week or more often; 2 = 4 times a week; 3 = 3 times a week; 4 = 2 times a week; 5 = once a week; 6 = less than once a week; 7 = I have a disability or a disease which does not enable me to exercise. Physical inactivity was defined as frequency <2 times/wk.
Inactive	1	

*Notes*: BMI = body mass index; CAIDE = Cardiovascular Risk Factors, Aging and Dementia; FINGER = Finnish Geriatric Intervention Study to Prevent Cognitive Impairment and Disability. CAIDE score for each FINGER participant was calculated by summing the number of points for the appropriate category of each of the risk factors. The score was calculated at baseline and 2-y visits.

### MRI Assessments

Prior to quantitative analysis, 3D T1-weighted and fluid-attenuated inversion recovery (FLAIR) images were visually inspected by a neuroradiologist. Participants scans were excluded if they had unexpected focal brain lesions and scanning issues potentially impacting volumetry e.g. no full brain coverage, artifacts, intensity inhomogeneity, and inadequate GM/white matter (WM) contrast. At each MRI site, regular phantom scans were performed, and quantitative measures of signal-to-noise ratio, uniformity, and geometric distortion were carried out.

Brain MRI scans were conducted for a subsample of 155 participants from 4 study sites of which 132 scans from 3 study sites passed quality control (12). Of these 132 participants, 112 were re-scanned in connection with the 2-year visit, and all scans passed quality control. Different MR systems were used, 1.5 T Avanto Siemens (3D-MPRAGE sequence, voxel size 1.2 × 1.2 × 1.2 mm, repetition time (TR) 2400 ms, echo time (TE) 3.5 ms, inversion time (TI) 1000 ms) at the Kuopio and Oulu sites, and 3T Ingenuity Philips (3D turbo field echo sequence [TFE] sequence, voxel size 1.0 × 1.0 × 1.0 mm, TR 8.1 ms, TE 3.7 ms) at the Turku site. Each site used the same scanner and imaging parameters for both baseline and 2-year scans.

Freesurfer (version 5.3, http://surfer.nmr.mgh.harvard.edu/) was used to measure regional brain volumes and cortical thicknesses. In case of geometric inaccuracy in boundaries between WM, GM, and cerebrospinal fluid (CSF) in the automated WM segmentation, manual editing was conducted. Brain volumes were normalized by the total intracranial volume (TIV) to account for between-person variations in head size (20).

WML volume was measured through the segmentation of WM hyperintensities (21). The method is based on the expectation -maximization algorithm. WML segmentation was done in 3 steps: (i) first, from T1 images, segmentation of WM into 2 classes representing hypointense and normal bright WM regions; (ii) second, using the results of the previous step as initialization, FLAIR images were segmented into 3 classes: CSF, normal brain tissue, and hyperintense voxels; (iii) third, using the results of the previous initialization step, WM and subcortical regions were segmented from the FLAIR images in 2 classes. The class with higher intensities was then regarded as the segmentation of WM hyperintensities (12).

### PiB-PET Assessments

PiB-PET scans were conducted in 48 participants in connection to the baseline FINGER visit, and 39 participants had a repeat scan in connection to the 2-year visit. PiB-PET was performed at the Turku trial site. [11C] PiB (N-methyl-[11 C]2-(4methylaminophenyl)-6-hydroxybenzothiazole) was produced as described earlier (22). On average, 406.3 (*SD* 107.7) MBq of PiB was injected intravenously and a scan from 60 to 90 min (3- × 10-min frames) after injection was performed with a Philips Ingenuity TF PET/MR scanner (Philips, Amsterdam, the Netherlands). A PiB composite score was calculated as the average of the prefrontal, parietal, lateral temporal, anterior cingulate, posterior cingulate, and precuneus regions (22). Region-based quantification was obtained as region to cerebellar cortex ratio over the 60- to 90-min scan duration.

### Statistical Analysis

The characteristics of the FINGER participants with 2 MRI or PET scans were compared between the intervention and control groups using *t* test or chi-squared test as appropriate. Analyses were done using Stata software version 12 (Stata Statistical Software: Release 12; StataCorp LP, College Station, TX). The level of statistical significance was *p* <.05 in all analyses.

For this post-hoc study, we chose 5 neuroimaging measurements (4 on MRI and 1 on PET) with clear established links to dementia/Alzheimer’s disease (AD). As all analyses are exploratory, that is, further testing and validation in larger studies will be needed, results for all 5 measurements are shown uncorrected for multiple testing. The following 4 MRI measures were considered: hippocampus volume, total GM volume, WML volume (all MRI volumes were divided by TIV), and a measure of cortical thickness in AD signature regions calculated as the average of cortical thickness in entorhinal, inferior temporal, middle temporal, and fusiform regions (23). The changes in CAIDE score, and MRI and PiB-PET measures were calculated as the difference between 2-year and baseline values, divided by time (in years).

After zero-skewness log-transformation for all the calculated change variables (hippocampus, total GM, and WML volume changes, AD signature thickness change and PIB composite change) that were not normally distributed, linear regression models were used to assess the associations between changes in each MRI measure or the PiB composite score (as dependent variables) and the change in CAIDE score. All models additionally included randomization group, Group × CAIDE score change interaction, site (except for the PiB composite score outcome, since PET scans were conducted at one site), and the corresponding baseline MRI or PET measure. We report standardized beta (β) coefficients and *p* values.

## Results

Characteristics of the FINGER participants with and without MRI or PiB-PET data at the study sites where brain scans were available were previously described (12). The MRI/PET population was not significantly different in demographic, clinical, and cognitive characteristics from the population without MRI/PET at these sites (12). Characteristics of the FINGER participants with 2 MRI or PET scans are presented in Tables 2 and 3. There were no significant differences between the intervention and control groups in the MRI/PET populations.

**Table 2.** Characteristics of the FINGER Participants With 2 MRI Scans

MRI Population Characteristics	Total (*N* = 112)	Intervention (*n* = 59)	Control (*n* = 53)	*p*
Baseline				
Age (y)	112	70.51 (4.86)	70.60 (4.64)	.85
Women, *n* (%)	112	24 (41)	30 (57)	.09
Education (y)	112	9.34 (2.96)	8.85 (2.13)	.32
Systolic blood pressure (mm Hg)	112	140.57 (15.73)	139.23 (14.71)	.64
Body mass index (kg/m^2^)	109	27.70 (3.76)	26.88 (3.52)	.24
Total cholesterol (mmol/L)	111	5.07 (1.03)	4.98 (0.91)	.64
Physically inactive, *n* (%)	108	13 (22)	9 (18)	.63
CAIDE Dementia Risk Score	104	7.76 (4–11)	7.27 (4–12)	.16
Total hippocampal volume (mL)^a^	112	7.21 (4.63–9.14)	7.05 (4.55–8.33)	.33
Total GM volume (mL)^a^	112	576.7 (443.4–667.3)	563.4 (406.3–709.6)	.18
WML volume (mL)^a^	100	11.88 (0.5–60.7)	11.71 (0.7– 74.4)	.95
Total intracranial volume (mL)^a^	112	1581.40 (1112.40–2039.10)	1524.4 (975.50–196.20)	.13
AD signature thickness (mm)^a^	112	2.77 (2.50–3.05)	2.77 (2.47–3.10)	.87
2-y visit				
Systolic blood pressure (mm Hg)	110	137.25 (16.50)	136.97 (15.53)	.92
Body mass index (kg/m^2^)	109	27.45 (3.64)	26.44 (3.66)	.14
Total cholesterol (mmol/L)	111	4.76 (0.93)	5.10 (1.01)	.07
Physically inactive, *n* (%)	108	8 (15.70)	8 (14.03)	.80
CAIDE Dementia Risk Score	107	7.64 (4–11)	7.27 (4–11)	.29
Total hippocampal volume (mL)^a^	112	7.03 (4.3–9.1)	6.83 (4.10–8.35)	.27
Total GM volume (mL)^a^	112	568.60 (434.44–670.4)	556.0 (414.82–698.55)	.19
WML volume (mL)^a^	100	13.6 (0.4–59.9)	13.0 (0.5–84.9)	.86
AD signature thickness (mm)^a^	112	2.73 (2.50–3.10)	2.75 (2.33–3.10)	.47

*Notes*: AD = Alzheimer’s disease signature (composite measure of entorhinal, inferior and middle temporal, and fusiform regions); CAIDE = Cardiovascular Risk Factors, Aging and Dementia; FINGER = Finnish Geriatric Intervention Study to Prevent Cognitive Impairment and Disability; GM = gray matter; MRI = magnetic resonance imaging; WML = white matter lesions. Values are means (*SD*) unless otherwise specified. MRI volumes presented in the table are not total intracranial volume-normalized. Differences between intervention and control groups were analyzed with chi-squared and *t* tests as appropriate.

^a^MRI and CAIDE values are mean (minimum–maximum), and MRI measures are based on longitudinal Freesurfer analyses.

**Table 3.** Characteristics of the FINGER Participants With 2 PiB-PET Scans

PiB-PET Population Characteristics	Total (*N* = 39)	Intervention (*n* = 18)	Control (*n* = 21)	*p*
Baseline				
Age (y)	39	72.40 (5.34)	72.06 (4.84)	.82
Women, *n* (%)	39	6 (33)	12 (57)	.13
Education (y)	39	9.05 (2.46)	8.95 (2.38)	.89
Systolic blood pressure (mm Hg)	39	140.25 (14.95)	136.76 (14.30)	.46
Body mass index (kg/m^2^)	39	27.20 (3.05)	25.80 (3.30)	.16
Total cholesterol (mmol/L)	38	5.21 (1.00)	5.02 (0.94)	.55
Physically inactive, *n* (%)	37	5 (28)	6 (32)	.80
CAIDE Dementia Risk Score	36	7.70 (1.61)	7.17 (2.31)	.45
PiB composite	39	1.52 (0.42)	1.60 (0.35)	.68
2-y visit				
Systolic blood pressure (mm Hg)	39	136.00 (15.76)	134.98 (17.84)	.85
Body mass index (kg/m^2^)	39	26.88 (3.11)	25.33 (3.41)	.14
Total cholesterol (mmol/L)	39	5.08 (0.94)	4.85 (0.97)	.45
Physically inactive, *n* (%)	39	5 (27.80)	5 (23.80)	.77
CAIDE Dementia Risk Score	39	7.70 (1.70)	6.95 (1.90)	.22
PiB composite	39	1.63 (0.50)	1.71 (0.39)	.59

*Notes*: CAIDE = Cardiovascular Risk Factors, Aging and Dementia; FINGER = Finnish Geriatric Intervention Study to Prevent Cognitive Impairment and Disability; PiB = Pittsburgh Compound B; PET = positron emission tomography. Values are means (*SD*) unless otherwise specified. Differences between intervention and control groups were analyzed with chi-squared and *t* tests as appropriate.

A reduction in CAIDE score was observed in 17 (30%) participants in the intervention, and 9 (21%) in the control group. Mean CAIDE score (*SD*) changed from 7.76 (1.70) to 7.64 (1.88) points in the intervention group but did not change in the control group (mean 7.27 points at both time points) (Table 2).

Overall, change in CAIDE score was not associated with change in imaging measures. However, there was a significant interaction between randomization group and change in CAIDE score (β coefficient = −0.40, *p* = .02). A reduction in the CAIDE score was associated with less pronounced decline in hippocampal volume in the intervention group (β coefficient = −0.27, *p* = .04), but not in the control group (β coefficient = 0.22, *p* = .19) (Table 4). Results were similar after additional adjustment for baseline age (continuous) and sex (Group × CAIDE score change interaction coefficient = −0.37, *p* = .03).

**Table 4.** Associations of CAIDE Dementia Risk Score Change With Change in Neuroimaging Markers

		Standardized β Coefficients (*p* value)		
Neuroimaging Measures	*N*	Intervention	Control	Randomization Group × CAIDE Score Change Interaction
Hippocampal volume	99	−0.27 (.04)	0.22 (.19)	−0.40 (.02)
Total gray matter volume	99	−0.007 (.96)	0.07 (.64)	−0.10 (.56)
WML volume	90	−0.01 (.94)	0.12 (.46)	−0.16 (.34)
AD signature thickness	99	0.10 (.47)	0.03 (.87)	0.06 (.72)
PiB-PET	36	0.05 (.82)	−0.25 (.30)	0.24 (.34)

*Notes*: AD = Alzheimer’s disease signature (composite measure of entorhinal, inferior and middle temporal, and fusiform regions); CAIDE = Cardiovascular Risk Factors, Aging and Dementia; PiB = Pittsburgh Compound B; PET = positron emission tomography; WML = white matter lesions. Values are standardized beta (β) coefficients (*p* values) from linear regressions with neuroimaging measures as dependent variables. Standardized β coefficients in the intervention and control groups are reported from the stratified analyses.

Given these findings, we conducted additional analyses using similar linear regression models, focusing on the change in individual components of the CAIDE score in relation to change in hippocampal volume. For systolic blood pressure, cholesterol, and BMI, the difference between 2-year and baseline values was divided by time (in years). Change in physical activity was dichotomized as increase vs no change/decrease in frequency from baseline to 2 years. The Randomization group × Change in physical activity interaction β coefficient was 0.30, *p* = .06, suggesting a trend for less pronounced decline in hippocampal volume with increasing physical activity levels. Changes in blood pressure, cholesterol, and BMI were not significantly associated with the change in hippocampal volume.

No significant associations were found between change in CAIDE score and change in other MRI measures or change in amyloid PiB-PET (Table 4).

## Discussion

In this exploratory FINGER neuroimaging substudy, a reduction in the CAIDE Dementia Risk Score during the intervention was associated with less decline in hippocampal volume. No associations were found between change in CAIDE score and changes in total GM or WML volume, cortical thickness in AD signature areas, or PiB composite score.

We have previously reported no significant differences between the intervention and control groups in change in MRI measures during the FINGER trial (24). However, the present study suggests that the intervention may have some benefits on hippocampal volume in individuals who succeed in improving their overall risk level as indicated by a reduction in CAIDE score. These findings are important since the hippocampus is known to be affected by neuronal loss during aging, and also early during the course of AD (25). Mean CAIDE score did not change in the control group. Thus, the observed association between reduction in CAIDE score and less decline in hippocampal volume in the intervention group indicates that benefits on structural brain changes may require more intensive modifications of an individual’s overall risk level.

Previous longitudinal observational studies focusing on risk prediction have reported links between higher baseline CAIDE score and several neuroimaging measures up to 30 years later, that is, lower hippocampal and GM volume, lower cortical thickness, more pronounced medial temporal atrophy (MTA), more pronounced WML, and higher WML volume, but not amyloid positivity on PiB-PET scans (11,12). Baseline CAIDE score has also been linked with longitudinal rates of brain atrophy in a middle aged population without dementia (6). Few other dementia risk scores have been tested in connection to dementia-related biomarkers in observational studies. A polygenic risk score has been longitudinally associated with cortical thinning in healthy adults (26). The Australian National University Alzheimer Disease Risk Index has been cross-sectionally associated with lower brain volumes (cortical GM and default mode network) but not the hippocampus in community-living individuals without dementia (27). However, no previous studies have investigated the change in CAIDE score (or any other dementia risk score) in relation to change in neuroimaging parameters, especially in the context of an intervention where emphasis is on prevention potential. Our findings are thus not directly comparable to previous studies, that is, it is possible that not all high-risk individuals in an observational, unselected cohort also have a high potential for prevention with a specific type of intervention.

Few longitudinal studies have so far investigated changes in individual risk factors for dementia in relation to changes in neuroimaging markers. The SMART-MR observational study reported that in patients with manifest arterial disease and higher baseline blood pressure, those with declining blood pressure levels over time had less progression of subcortical atrophy compared to those with increasing blood pressure levels (28). Another observational study linked small increases in blood pressure over time with increased brain atrophy and subcortical lesions 5 years later (29). However, a small intervention study reported that successful treatment of blood pressure was not associated with regional GM volume (30). The multidomain vascular intervention Prevention of dementia by intensive vascular care (pre DIVA), targeting older individuals from general population, did not decrease WM hyperintensities accumulation over 3 years. However, better intervention effects were observed in those with higher baseline WM hyperintensities volumes (31). The SPRINT trial reported significantly less increase in cerebral WML for intervention targeting systolic blood pressure <120 mm Hg, compared to systolic blood pressure <140 mm Hg. However, no significant difference in total brain volume change were reported for either of the groups (32).

Studies linking other risk factors with brain structure have reported mixed results. Decreased levels of high-density lipoprotein (HDL) have been associated with GM reductions in adults with normal cognition (33). Lowering of total cholesterol in older patients undergoing antihypertensive and statin therapy has been reported to reduce progression of WM hyperintensities (34,35). However, HDL cholesterol levels were not related to hippocampal volume in the Rotterdam scan study (36) contrary to the findings of Wolf et al. reporting an association (37). Increase in BMI over time has been associated with cortical thinning at midlife which continued in the late life. Decreasing BMI in late life has also been related to cortical thinning (38). Several intervention studies have reported gains in hippocampal volume in response to physical activity (39–41).

Although the impact of the CAIDE score reduction on less decline in hippocampal volume in the present study may at least partly be explained by increasing levels of physical activity, we did not find significant associations between change in other individual risk factors included in the CAIDE score (blood pressure, BMI, total cholesterol) and change in neuroimaging parameters. As a weighted combination of several risk factors for dementia, the CAIDE score may be a better reflection of the overall impact of these factors taken together. The change in CAIDE score over time may also be a more accurate indicator of the impact of complex lifestyle modifications on the overall dementia risk. Different persons with the same overall risk can have different risk factor profiles. Depending on which factors make up a person’s specific profile, a reduction in overall risk can mean that different factors may change in different persons.

The main strengths of the present study are its randomized controlled design with 2-year longitudinal neuroimaging data that are not very common in lifestyle intervention studies. While studies investigating associations between dementia risk scores and dementia-related biomarkers have mostly been observational, the current study focused on change over time in both CAIDE score and neuroimaging markers in the context of an intervention that has previously shown significant benefits on cognition (15). Also, the FINGER trial included an at-risk population without substantial cognitive impairment, which may have more “room for improvement” for dementia risk modification.

The relatively small size of the FINGER neuroimaging population is the most important limitation of this study. As all analyses were post hoc, findings from the present study must be regarded as exploratory and will need to be verified in larger studies. For this post-hoc study, we chose 5 neuroimaging measurements with clear established links to dementia/AD. Results for all 5 measurements are shown uncorrected, since multiple comparison correction does not change the exploratory nature of the study. No interpretation can be currently made regarding potential effect size, that is, how much decrease in overall risk would be needed to see an impact on brain structure, and how this would affect future dementia development. FINGER participants are representative for the at-risk segment of the Finnish general population (42), but validation is needed in other, preferably multi-ethnic populations. Also, the neuroimaging subgroup in this analysis may not be representative of all FINGER trial participants (12). Another limitation is that different FINGER trial sites used different MRI scanners. To account for this, we adjusted all analyses for study site. Moreover, as reported previously, Freesurfer morphometric procedures have shown good test–retest reliability across scanner manufacturers and across field strengths (43,44).

In conclusion, a reduction in the overall dementia risk profile as indicated by the CAIDE score change initiated during the intervention was related to benefits on hippocampal volume. The CAIDE Dementia Risk Score is a simple and practical tool not only for estimating dementia risk but also for quantifying prevention potential. However, considering that this score is based on simple cutoffs for risk factors, it is important to develop dementia risk estimation tools that are even more sensitive to capturing lifestyle changes, and their potential impact on brain structure.

## References

[1] World Health Organization. Risk Reduction of Cognitive Decline and Dementia: WHO Guidelines. Geneva, Switzerland: World Health Organization; 2019. 31219687

[2] van Middelaar T , Hoevenaar-Blom MP, van Gool WA, et al. Modifiable dementia risk score to study heterogeneity in treatment effect of a dementia prevention trial: a post hoc analysis in the preDIVA trial using the LIBRA index. Alzheimers Res Ther. 2018;10:62. doi:10.1186/s13195-018-0389-4 29960597 PMC6026510

[3] Hou XH , Feng L, Zhang C, Cao XP, Tan L, Yu JT. Models for predicting risk of dementia: a systematic review. J Neurol Neurosurg Psychiatry. 2019;90:373–379. doi:10.1136/jnnp-2018-318212 29954871

[4] Kivipelto M , Ngandu T, Laatikainen T, Winblad B, Soininen H, Tuomilehto J. Risk score for the prediction of dementia risk in 20 years among middle aged people: a longitudinal, population-based study. Lancet Neurol. 2006;5:735–741. doi:10.1016/S1474-4422(06)70537-3 16914401

[5] Exalto LG , Quesenberry CP, Barnes D, Kivipelto M, Biessels GJ, Whitmer RA. Midlife risk score for the prediction of dementia four decades later. Alzheimers Dement. 2014;10:562–570. doi:10.1016/j.jalz.2013.05.1772 24035147

[6] O’Brien JT , Firbank MJ, Ritchie K, et al. Association between midlife dementia risk factors and longitudinal brain atrophy: the PREVENT-dementia study. J Neurol Neurosurg Psychiatry. 2020;91:158–161. doi:10.1136/jnnp-2019-321652 31806724

[7] Ecay-Torres M , Estanga A, Tainta M, et al. Increased CAIDE dementia risk, cognition, CSF biomarkers, and vascular burden in healthy adults. Neurology. 2018;91:e217–e226. doi:10.1212/WNL.0000000000005824 29898969

[8] Enache D , Solomon A, Cavallin L, et al. CAIDE Dementia Risk Score and biomarkers of neurodegeneration in memory clinic patients without dementia. Neurobiol Aging. 2016;42:124–131. doi:10.1016/j.neurobiolaging.2016.03.007 27143429

[9] Kaffashian S , Dugravot A, Elbaz A, et al. Predicting cognitive decline: a dementia risk score vs. the Framingham vascular risk scores. Neurology. 2013;80:1300–1306. doi:10.1212/WNL.0b013e31828ab370 23547265 PMC3656460

[10] Reijmer YD , van den Berg E, van Sonsbeek S, et al. Dementia risk score predicts cognitive impairment after a period of 15 years in a nondemented population. Dement Geriatr Cogn Disord. 2011;31:152–157. doi:10.1159/000324437 21335972

[11] Vuorinen M , Spulber G, Damangir S, et al. Midlife CAIDE dementia risk score and dementia-related brain changes up to 30 years later on magnetic resonance imaging. J Alzheimers Dis. 2015;44:93–101. doi:10.3233/JAD-140924 25190628

[12] Stephen R , Liu Y, Ngandu T, et al. Associations of CAIDE Dementia Risk Score with MRI, PIB-PET measures, and cognition. J Alzheimers Dis. 2017;59:695–705. doi:10.3233/JAD-170092 28671114 PMC5523839

[13] Hooshmand B , Polvikoski T, Kivipelto M, et al. CAIDE Dementia Risk Score, Alzheimer and cerebrovascular pathology: a population-based autopsy study. J Intern Med. 2018;283:597–603. doi:10.1111/joim.12736 29411449

[14] Coley N , Hoevenaar-Blom MP, van Dalen JW, et al. Dementia risk scores as surrogate outcomes for lifestyle-based multidomain prevention trials—rationale, preliminary evidence and challenges. Alzheimer’s Dement. 2020;16:1674–1685. doi:10.1002/alz.12169 32803862

[15] Ngandu T , Lehtisalo J, Solomon A, et al. A 2 year multidomain intervention of diet, exercise, cognitive training, and vascular risk monitoring versus control to prevent cognitive decline in at-risk elderly people (FINGER): a randomised controlled trial. Lancet. 2015;385:2255–2263. doi:10.1016/S0140-6736(15)60461-5 25771249

[16] Solomon A , Levälahti E, Antikainen R, et al. Effects of a multidomain lifestyle intervention on overall risk for dementia: the finger randomized controlled trial. Alzheimer’s Dementia. 2018;14(7):P1024–P1025. https://www.alzheimersanddementia.com/article/S1552-5260(18)32970–4/abstract

[17] Kivipelto M , Solomon A, Ahtiluoto S, et al. The Finnish Geriatric Intervention Study to Prevent Cognitive Impairment and Disability (FINGER): study design and progress. Alzheimers Dement. 2013;9:657–665. doi:10.1016/j.jalz.2012.09.012 23332672

[18] National Nutrition Council. Finnish Nutrition Recommendations—Diet and Physical Activity in Balance. Edita Publishing Ltd; 2005.

[19] Dahlin E , Neely AS, Larsson A, Bäckman L, Nyberg L. Transfer of learning after updating training mediated by the striatum. Science. 2008;320:1510–1512. doi:10.1126/science.1155466 18556560

[20] Whitwell JL , Crum WR, Watt HC, Fox NC. Normalization of cerebral volumes by use of intracranial volume: implications for longitudinal quantitative MR imaging. AJNR Am J Neuroradiol. 2001;22:1483–1489. 11559495 PMC7974589

[21] Wang Y , Catindig JA, Hilal S, et al. Multi-stage segmentation of white matter hyperintensity, cortical and lacunar infarcts. Neuroimage. 2012;60:2379–2388. doi:10.1016/j.neuroimage.2012.02.034 22387175

[22] Kemppainen NM , Aalto S, Wilson IA, et al. Voxel-based analysis of PET amyloid ligand [11C]PIB uptake in Alzheimer disease. Neurology. 2006;67:1575–1580. doi:10.1212/01.wnl.0000240117.55680.0a 16971697

[23] Jack CR, Jr., Wiste HJ, Weigand SD, et al. Different definitions of neurodegeneration produce similar amyloid/neurodegeneration biomarker group findings. Brain. 2015;138(Pt 12):3747–3759. doi:10.1093/brain/awv283 26428666 PMC4655341

[24] Stephen R , Liu Y, Ngandu T, et al. Brain volumes and cortical thickness on MRI in the Finnish Geriatric Intervention Study to Prevent Cognitive Impairment and Disability (FINGER). Alzheimers Res Ther. 2019;11:53. doi:10.1186/s13195-019-0506-z 31164160 PMC6549301

[25] Dubois B , Feldman HH, Jacova C, et al. Advancing research diagnostic criteria for Alzheimer’s disease: the IWG-2 criteria. Lancet Neurol. 2014;13:614–629. doi:10.1016/S1474-4422(14)70090-0 24849862

[26] Harrison TM , Mahmood Z, Lau EP, et al. An Alzheimer’s disease genetic risk score predicts longitudinal thinning of hippocampal complex subregions in healthy older adults. eNeuro. 2016;3(3):795–804. doi:10.1523/ENEURO.0098-16.2016 PMC4945997 27482534

[27] Cherbuin N , Shaw ME, Walsh E, Sachdev P, Anstey KJ. Validated Alzheimer’s Disease Risk Index (ANU-ADRI) is associated with smaller volumes in the default mode network in the early 60s. Brain Imaging Behav. 2019;13(1):65–74. doi:10.1007/s11682-017-9789-5 29243120 PMC6409311

[28] Jochemsen HM , Muller M, Visseren FL, et al. Blood pressure and progression of brain atrophy: the SMART-MR Study. JAMA Neurol. 2013;70:1046–1053. doi:10.1001/jamaneurol.2013.217 23753860

[29] Goldstein IB , Bartzokis G, Guthrie D, Shapiro D. Ambulatory blood pressure and the brain: a 5-year follow-up. Neurology. 2005;64:1846–1852. doi:10.1212/01.WNL.0000164712.24389.BB 15955932

[30] Jennings JR , Mendelson DN, Muldoon MF, et al. Regional grey matter shrinks in hypertensive individuals despite successful lowering of blood pressure. J Hum Hypertens. 2012;26:295–305. doi:10.1038/jhh.2011.31 21490622 PMC3137674

[31] van Dalen JW , Moll van Charante EP, Caan MWA, et al. Effect of long-term vascular care on progression of cerebrovascular lesions: magnetic resonance imaging substudy of the PreDIVA trial (Prevention of Dementia by Intensive Vascular Care). Stroke. 2017;48:1842–1848. doi:10.1161/STROKEAHA.117.017207 28596452

[32] Nasrallah IM , Pajewski NM, Auchus AP, et al. Association of intensive vs standard blood pressure control with cerebral white matter lesions. J Am Med Assoc. 2019;322(6):524–534. doi:10.1001/jama.2019.10551 PMC6692679 31408137

[33] Ward MA , Bendlin BB, McLaren DG, et al. Low HDL cholesterol is associated with lower gray matter volume in cognitively healthy adults. Front Aging Neurosci. 2010;2:29. doi:10.3389/fnagi.2010.00029 20725527 PMC2914583

[34] Ji T , Zhao Y, Wang J, et al. Effect of low-dose statins and apolipoprotein E genotype on cerebral small vessel disease in older hypertensive patients: a subgroup analysis of a randomized clinical trial. J Am Med Dir Assoc. 2018;19:995–1002.e4. doi:10.1016/j.jamda.2018.05.025 30006015

[35] Zhang H , Cui Y, Zhao Y, et al. Effects of sartans and low-dose statins on cerebral white matter hyperintensities and cognitive function in older patients with hypertension: a randomized, double-blind and placebo-controlled clinical trial. Hypertens Res. 2019;42:717–729. doi:10.1038/s41440-018-0165-7 30552406

[36] den Heijer T , Hofman A, Koudstaal PJ, et al. Serum lipids and hippocampal volume: the link to Alzheimer’s disease? Ann Neurol. 2005;57(5):779–780; author reply 7780. doi:10.1002/ana.20469 15852390

[37] Wolf H , Hensel A, Arendt T, et al. Serum lipids and hippocampal volume: the link to Alzheimer’s disease? Ann Neurol. 2004;56(5):745–748. doi:10.1002/ana.20289 15505826

[38] Shaw ME , Sachdev PS, Abhayaratna W, Anstey KJ, Cherbuin N. Body mass index is associated with cortical thinning with different patterns in mid- and late-life. Int J Obes (Lond). 2018;42:455–461. doi:10.1038/ijo.2017.254 28993708

[39] Morris JK , Vidoni ED, Johnson DK, et al. Aerobic exercise for Alzheimer’s disease: a randomized controlled pilot trial. PLoS One. 2017;12:e0170547. doi:10.1371/journal.pone.0170547 28187125 PMC5302785

[40] Rosano C , Guralnik J, Pahor M, et al. Hippocampal response to a 24-month physical activity intervention in sedentary older adults. Am J Geriatr Psychiatry. 2017;25:209–217. doi:10.1016/j.jagp.2016.11.007 27986412 PMC5568026

[41] ten Brinke LF , Bolandzadeh N, Nagamatsu LS, et al. Aerobic exercise increases hippocampal volume in older women with probable mild cognitive impairment: a 6-month randomised controlled trial. Br J Sports Med. 2015;49(4):248–254. doi:10.1136/bjsports-2013-093184 24711660 PMC4508129

[42] Ngandu T , Lehtisalo J, Levälahti E, et al. Recruitment and baseline characteristics of participants in the Finnish Geriatric Intervention Study to Prevent Cognitive Impairment and Disability (FINGER)—a randomized controlled lifestyle trial. Int J Environ Res Public Health. 2014;11:9345–9360. doi:10.3390/ijerph110909345 25211775 PMC4199023

[43] Han X , Jovicich J, Salat D, et al. Reliability of MRI-derived measurements of human cerebral cortical thickness: the effects of field strength, scanner upgrade and manufacturer. Neuroimage. 2006;32:180–194. doi:10.1016/j.neuroimage.2006.02.051 16651008

[44] Reuter M , Schmansky NJ, Rosas HD, Fischl B. Within-subject template estimation for unbiased longitudinal image analysis. Neuroimage. 2012;61:1402–1418. doi:10.1016/j.neuroimage.2012.02.084 22430496 PMC3389460

